# Rapid On-Site Detection of Arboviruses by a Direct RT-qPCR Assay

**DOI:** 10.3390/bios13121035

**Published:** 2023-12-16

**Authors:** Moufid Mhamadi, Giulia Mencattelli, Alioune Gaye, El Hadji Ndiaye, Aïssatou Aïcha Sow, Martin Faye, Marie Henriette Dior Ndione, Moussa Moïse Diagne, Moundhir Mhamadi, Ousmane Faye, Manfred Weidmann, Oumar Faye, Mawlouth Diallo, Cheikh Tidiane Diagne

**Affiliations:** 1 Department of Virology, Fondation Institut Pasteur de Dakar 36, Avenue Pasteur, Dakar 220, Senegal; giulia.mencattelli@unitn.it (G.M.); aicha.sow@inrs.ca (A.A.S.); martin.faye@pasteur.sn (M.F.); marie.ndione@pasteur.sn (M.H.D.N.); moussamoise.diagne@pasteur.sn (M.M.D.); ousmane.faye@pasteur.sn (O.F.); oumar.faye@pasteur.sn (O.F.); 2 Department of Medical Zoology, Fondation Institut Pasteur de Dakar 36, Avenue Pasteur, Dakar 220, Senegal; alioune.gaye@pasteur.sn (A.G.); elhadji.ndiaye@pasteur.sn (E.H.N.); 3 DIATROPIX, Fondation Institut Pasteur de Dakar 36, Avenue Pasteur, Dakar 220, Senegal; moundhir.mhamadi.km@gmail.com (M.M.); mawlouth.diallo@pasteur.sn (M.D.); 4 Institute of Microbiology and Virology, Brandenburg Medical School Theodor Fontane, 01968 Senftenberg, Germany; manfred.weidmann@mhb-fontane.de

**Keywords:** direct RT-qPCR, supernatant, arbovirus, infected mosquitoes, field diagnosis

## Abstract

Arthropod-borne diseases currently constitute a source of major health concerns worldwide. They account for about 50% of global infectious diseases and cause nearly 700,000 deaths every year. Their rapid increase and spread constitute a huge challenge for public health, highlighting the need for early detection during epidemics, to curtail the virus spread, and to enhance outbreak management. Here, we compared a standard quantitative polymerase chain reaction (RT-qPCR) and a direct RT-qPCR assay for the detection of Zika (ZIKV), Chikungunya (CHIKV), and Rift Valley Fever (RVFV) viruses from experimentally infected-mosquitoes. The direct RT-qPCR could be completed within 1.5 h and required 1 µL of viral supernatant from homogenized mosquito body pools. Results showed that the direct RT-qPCR can detect 85.71%, 89%, and 100% of CHIKV, RVFV, and ZIKV samples by direct amplifications compared to the standard method. The use of 1:10 diluted supernatant is suggested for CHIKV and RVFV direct RT-qPCR. Despite a slight drop in sensitivity for direct PCR, our technique is more affordable, less time-consuming, and provides a better option for qualitative field diagnosis during outbreak management. It represents an alternative when extraction and purification steps are not possible because of insufficient sample volume or biosecurity issues.

## 1. Introduction

Vector-borne viruses are a group of viruses widely circulating the World [1]. Due to their ability to spread across new areas, they constitute a serious public health concern in developing as well as developed countries [2,3]. Vector-borne diseases cause about 1 billion cases and, Chikungunya virus (CHIKV) [4,5], Zika virus (ZIKV) [6], and Rift Valley Fever virus (RVFV) [7,8] cause 0.044%, 0.0022%, and 0.00022% of these cases, respectively. These viruses transmitted mainly by mosquito bites, are considered important human and veterinary pathogens that can lead to lethal illness and severe socio-economic consequences [9,10,11].

The CHIKV, a member of the Semliki Forest antigenic group, belongs to the genus *Alphavirus* in the *Togaviridae* family. Its genome is an 11.8 Kb single-strand positive ribonucleic acid (RNA), with a 5′ 7-methylguanosine cap and a 3′ poly-A tail. It has an enveloped genome of 70 nm, carrying two overlapping open reading frames (ORFs) separated by an untranslated region (5′UTR3′) and a non-coding junction. The ORFs encode for five structural proteins (capsid, E1, E2, E3, and 6K) and four non-structural proteins (nsP1 to nsP4) [12,13]. *Aedes* mosquitoes are the most important CHIKV vectors, while non-human primates are the main viral reservoirs. In infected humans, the virus induces symptoms such as high fever, moderate–severe arthralgia, and myalgia [5]. First documented in Tanzania in 1952, the virus has since then spread in many countries worldwide, with reported outbreaks in Africa, Asia, Europe, and North and South America [12,13].

The ZIKV is a mosquito-borne virus belonging to the family *Flaviviridae* in the genus *Flavivirus*. Like other *Flaviviruses*, ZIKV is a 50 nm enveloped virus constituted by an inner nucleocapsid and an outer lipid bilayer. The 10.8 Kb viral RNA genome contains a single ORF flanked by 3′ and 5′ non-coding regions [14]. The ORF encodes for a large polyprotein constituted by three structural proteins (C, prM, and E) and seven non-structural proteins (NS1, NS2A, NS2B, NS3, NS4A, NS4B, and NS5). ZIKV was isolated from a rhesus monkey in Uganda in 1947. In 2007, this virus caused the first major outbreak in the Yap Islands of Micronesia [15]. Since then, it spread across Pacific islands in 2013–2014 [16,17], reached Latin America in 2013–2015 [18], and ended up affecting more than 30 countries in the Americas [14]. ZIKV is maintained in nature in a sylvatic cycle between non-human primates and *Aedes* mosquitoes, the latter considered the most important viral vector. It is known to cause mild symptoms, even since the 2013–2015 Latin America outbreak, Zika fever has been associated with Guillain Barré syndrome in adults and microcephaly in neonate humans [6,19]. The sexual and vertical transmission of ZIKV in humans were also documented [19,20].

The RVFV is a *Phlebovirus* belonging to the family *Phenuviridae* in the *Bunyavirales* order. It is an enveloped virus of 110 nm with a negative tri-segmented RNA genome of 11.5 Kb, including a large (L), medium (M), and small (S) segment [21]. The L segment encodes for an RNA-dependent RNA polymerase, the M segment for two glycoproteins, Gc and Gn, and one non-structural protein (NSm), and the S segment for an open reading form (ORF) and one non-structural protein (NSs) [21]. Since the first report of RVFV in Kenya in 1931, the virus has been reported widely in Africa and the Arabian peninsula, causing many outbreaks in livestock and humans [22]. The virus is mainly transmitted by mosquito bites (*Aedes* and *Culex* mosquitoes), but also by contact with infected tissues and aerosols. The disease incidence often increases during the rainy season, when mosquitoes are abundant and most active [23].

Laboratory diagnosis relies on virus isolation by cellular culture or detection of the virus-specific RNA through reverse transcription and quantitative polymerase chain reaction (RT-qPCR) [24]. However, these techniques are time- and cost-consuming and demand stable laboratory settings [25,26]. In particular, RT-qPCR, considered a gold standard for detecting the RNA of arboviruses and shows high efficiency due to its sensitivity and specificity [27]; however, it requires an initial RNA extraction. The RNA extraction from cell culture supernatants, either with the automated Magna Pure 96 system (Roche, Penzberg, Germany) or with viral column-based methods, such as an RNA mini kit (QIAGEN, Hilden, Germany), is technologically difficult and often not available in remote or rural areas. It is expensive and time-consuming, and it requires experienced technicians as well as standard laboratory conditions [28]. Thus, an alternative technique is required.

Recently, the worldwide spread of a Coronavirus firstly reported in China, namely SARS-CoV-2, in December 2019, led to an alarming worldwide shortage of viral RNA extraction kits, necessitating new simple and reliable procedure for direct RNA amplification without prior extraction [29]. 

The development of a rapid, sensible, and accurate technique to detect infectious pathogens by direct real-time amplification without prior nucleic acid (NA) extraction would allow for the simplified rapid detection and monitoring of viral circulation in hosts as well as mosquitoes in the field, potentially improving outbreak management. Rosenstierne, in 2020, showed the possibility of performing NA amplification without prior SARS-CoV-2 RNA extraction by initially heating the sample at 98 °C for 5 min as an alternative to the MagNA pure purification step [29]. Lang Li et al. described a ZIKV direct RT-qPCR assay conducted on saliva, serum, throat swabs, whole blood, and urine [30]. However, while the heating process chosen by Fomsgaard and Rosenstierne may lead to RNA degradation, the Lang Li et al. technique showed high sensitivity and specificity but was not tested on experimentally infected mosquitoes with diverse arboviruses. In this study, we tested the feasibility of a direct RT-qPCR analysis without prior RNA extraction and purification on mosquitoes experimentally infected with three arboviruses (ZIKV, CHIKV, and RVFV), mimicking typical field conditions. This simple, and fast workflow can be used as an alternative in public health and diagnostic laboratories as well as in the mobile field setting to allow for the rapid molecular detection of arboviruses during epidemics.

## 2. Materials and Methods

### 2.1. Mosquito Population Sampling

Mosquito sampling was undertaken in three different bioclimatic areas of Senegal: Kedougou (12°33′00″ N, 12°11′00″ W), located in the Sudano–Guinean region, Dakar (14°43′29″ N, 17°28′24″ W), located in the Sahelo–Sudanian region, and Barkedji (15°17 North, 14°53 West), located in the Sahelian savannah region. *Aedes (Ae.) vexans*, a mosquito species most frequently associated with RVFV in Senegal, was collected in Barkedji, a bioclimatic zone where several entomological studies have been conducted on RVFV vectors for many years [7,31,32]. *Ae. aegypti*, anthropophilic mosquitoes frequently associated with CHIKV and ZIKV [33,34], were collected in the Kedougou and Dakar regions. Larvae and pupae were collected from the field, while adult mosquitoes were reared in the laboratory at a temperature of 26–28 °C, relative humidity of 70–75%, and a light–dark photoperiod of 12:12 h. To obtain F1 generation eggs, the F0 generation female adult mosquitoes were frequently fed on guinea pigs. Larvae hatched from the obtained eggs were reared into F1 generation adults. Three-to-five-day-old F1 generation adult mosquitoes used for the experimental infection were reared exclusively with a 10% sucrose solution under the laboratory conditions described above [33,35,36].

### 2.2. Virus Strain and Viral Stock Preparations

Three virus isolates corresponding to ZIKV, CHIKV, and RVFV were used for experimental infections. The virus strains used and their origin, place, year of isolation, and passage history are shown in Table 1.

All virus strains were passed one time in *Ae. albopictus* continuous cell line (C6/36), initially provided by the American Type Culture Collection (ATCC). Briefly, C6/36 cells were cultured in Leibovitz-15 (L-15) medium (GibcoBRL, Grand Island, NY, USA) supplemented with 10% fetal bovine serum (FBS) (Gibco BRL, Grand Island, NY, USA), 10% of Bacto™ Tryptose Phosphate Broth (Thermofisher, Waltham, MA, USA), and 1% penicillin–streptomycin solution (Thermofisher, Waltham, MA, USA), and maintained at 28 °C in 25 cm^2^ tissue culture flasks. After medium removal from the flasks, 150 uL of the viral supernatant solution was added directly to the C6/36 cellular monolayers. C6/36 cells were then left for 1 h incubation at room temperature. After 1 h, 5 mL of L-15 medium supplemented with 5% FBS was added to the infected cells that were incubated at 28 °C. After 7 days of incubation, infected cells’ supernatants were harvested and cells were analyzed by indirect immunofluorescence assay (IFA) using specific immune ascites of each virus to assess the infection [37]. The infected cell supernatants were aliquoted, frozen at −80 °C, and used as viral stocks for mosquito infections. Subsequent stocks of CHIKV, ZIKV, and RVFV were determined by plaque forming unit assay using Porcine stable kidney cell line (PS cells, American Type Culture Collection, Manassas, VA, USA) [38]. Due to the co-infection of the ZIKV supernatant with a CHIKV strain, we used an indirect immunofluorescence assay as an alternative to the standard plaque assay for their titration [26,39].

### 2.3. Mosquitoes’ Oral Infection

Three-tofive-day-old F1 generations of female mosquitoes were placed into 0.45 L cardboard cages and starved for 24 h before being allowed to feed on a nutritive solution through a membrane feeder placed on top of their cage [36,40]. More precisely, the infectious meal consisted of 33% rabbit erythrocytes washed one time with 1% phosphate-buffered saline, 33% (*v*/*v*) virus stock (either CHIKV, ZIKV or RVFV) suspended in Leibovitz 15 (L15) cell culture medium, 20% (*v*/*v*) fetal bovine serum, 1% sucrose, and 5 mM ATP added as a phagostimulant. The membrane feeder was maintained at 37 °C and mosquitoes were allowed to feed for 60 min. After feeding, fully engorged mosquitoes were coldly anesthetized, transferred to 1 L cardboard cages with a net on top, and maintained with 10% sucrose at 27 °C, relative humidity of 80%, and a light/dark photoperiod of 16:8 h for the extrinsic incubation of the virus during 15 days. One experiment per virus strain was performed. *Ae. vexans* were orally infected with RVFV, while *Ae. aegypti* were infected with CHIKV or ZIKV, as shown in previous vector competence studies [32,34,41]. For each infection experiment, a sample of the virus–blood suspension was collected at the end of the mosquito feeding for virus titration.

### 2.4. Mosquito Processing and Virus Detection

At 15 days post-infection (dpi), mosquitoes were coldly anesthetized, and each mosquito head and body (whole body with legs and wings) samples were placed in distinct 2 mL Eppendorf tubes. Mosquito heads were separately triturated in 500 μL of L-15 cell culture medium (GibcoBRL, Grand Island, NY, USA) complemented with 20% FBS. To separate the virus supernatant from the mosquito’s debris, each homogenate was centrifugated for 10 min at 10,000 rpm at 4 °C. Viral RNA extraction was then conducted on the virus supernatant using the QIAamp Viral RNA miniKit (QIAgen, Heiden, Germany), according to the manufacturer’s instructions. And purified RNA was eluted in 60 µL of elution buffer AVE. RNAs extracted from each engorged mosquito head were tested by real-time RT-qPCR using QuantiTect Probe RT-PCR Kit (200) (Qiagen Inc., Santa Clarita, CA, USA) on the thermocycler ABI Prism 7500 SDS (Applied Biosystems, Foster City, CA, USA). Standard RT-qPCR was performed according to the manufacturer’s recommendations. For this, 5 µL of extracted RNA were mixed with 10 μL of 2× QuantiTect Probe RT-PCR Master Mix, 6.8 μL of RNase-free water, 1.25 μL of each primer (CHIKV [42], RVFV [43], and ZIKV [44]) at 10 μM, 0.5 μL of probe at 10 μM (CHIKV [42], RVFV [43], and ZIKV [44]), and 0.2 μL QuantiTect RT Mix (Omniscript^®^ Reverse Transcriptase and Sensiscript^®^ Reverse Transcriptase) to a total volume of 25 μL. On an ABI Prism 7500 SDS (Applied Biosystems, Foster City, CA, USA) thermocycler, the following cycle conditions were used: RT step at 50.0 °C for 10 min, 95.0 °C for 15 min, 40 cycles of 15 s at 95.0 °C, and 1 min at 60 °C. Following the testing of the heads, the bodies corresponding to the positive heads were selected, pooled, and used for the direct quantitative polymerase chain reaction (direct RT-qPCR) assay. Then, these samples were tested by standard RT-qPCR and direct quantitative polymerase chain reaction (direct RT-qPCR) to compare the sensitivity and specificity of both techniques. For each virus, pools of mosquito bodies were made by combining positive mosquito bodies with negative mosquito bodies from the insectarium of IPD to mime field reality. These negative mosquitoes are mosquitoes from the IPD insectarium that have never been exposed to the viruses of interest.

### 2.5. Direct RT-PCR Assay

Whole supernatant from body pools was used for direct RT-qPCR assay. The direct RT-qPCR protocol uses two different parameters: for one, we used 1 µL of body pool supernatant, and for the other, we used 1 µL of body pool supernatant diluted at 1/10 in nuclease-free water. Next, 1 µL of body pool supernatant (diluted or not) was added in 10 μL of buffer (2× QuantiTect Probe RT-PCR Master Mix), 10.8 μL of RNase free water, 1.25 μL of each primer (CHIKV [42], RVFV [43], and ZIKV [44], cf. Table 2) at 10 μM, 0.5 μL of probe at 10 μM (CHIKV [42], RVFV [43], and ZIKV [44] cf. Table 2), and 0.2 μL of QuantiTect RT-Mix (Omniscript^®^ Reverse Transcriptase and Sensiscript^®^ Reverse Transcriptase) to a total volume of 25 μL. The RT-qPCR was performed using an ABI Prism 7500 SDS (Applied Biosystems, Foster City, CA, USA). The cycling conditions were RT step at 50.0 °C for 10 min, 95.0 °C for 15 min, 40 cycles of 15 s at 95.0 °C, and 1 min at 60 °C.

### 2.6. Data Analysis

Mosquito samples were considered positive when they were detected by RT-qPCR with Cq value of <40. Cq values ≥ 40 were included in the study as negative results. The mean infection rates between extracted RNA, pure supernatant, and diluted supernatant were analyzed by Student’s *t*-test and linear regression test. *p*-values (denoted *p*) are provided. *p*-Values < 0.05 were considered as significant. Sensitivity, specificity, and accuracy were calculated using R software 4.3.1. The validity of the *t*-test was verified using the Jarque Bera test and Fisher test for testing the normality and variance equality, respectively.

## 3. Results

A total of 82 *Ae. aegypti*, 70 *Ae. aegypti*, and 63 *Ae. vexans* mosquitoes were engorged with CHIKV, ZIKV, and RVFV infectious blood meal, respectively. The viral load was about the same as before the blood meal for ZIKV (1 *×* 10^7^ PFU/mL), CHIKV (1.5 *×* 10^7^ PFU/mL), and RVFV (2 *×* 10^7^ PFU/mL) viruses. After RNA extraction and amplification, 36, 45, and 45 mosquito heads were found to be positive for CHIKV, ZIKV, and RVFV, respectively. Pools composed of mosquito bodies corresponding to viral RNA specific-PCR positive heads were made as described in Table 3. Of note, standard and direct RT-PCR was performed on mosquito body pool supernatants.

Unfortunately, while 10 mosquito body pools were made for ZIKV and RVFV experiments, only 9 mosquito body pools could be made for the CHIKV experiments because of an insufficient number of infected mosquitoes. A total of 400 μL of L-15 cell culture medium (GibcoBRL, Grand Island, NY, USA) containing 10% FBS was added to each pool. Homogenization and centrifugation processes were performed as previously described.

### 3.1. Chikungunya Virus

CHIKV results highlighted an average difference sensitivity of 5.9 Cq value units in Cq values between the standard and the direct RT-qPCR, and of 5.2 Cq value units between the standard and the direct 1:10 diluted supernatant RT-qPCR. The standard RT-qPCR enabled CHIKV detection in seven pools of mosquito bodies (PM1–PM7), while CHIKV was detected in four pools (PM1–PM4) and six pools (PM1–PM6) with the direct RT-qPCR on pure supernatant and 1/10 diluted supernatant, respectively (Table 4).

Interestingly, the mean difference between non-extracted pure supernatant and non-extracted 1/10 diluted supernatant was not significant (*p* = 0.39). However, the use of 1:10 diluted supernatant for direct amplification allowed for CHIKV detection in two additional pools compared to pure supernatant (PM6 and PM7) (Table 5).

### 3.2. Rift Valley Fever Virus

The RVFV standard and direct RT-qPCR data highlighted an average sensitivity difference of 8.9 Cq value units (*p* = 0.004) in Cq values between extracted RNA and non-extracted pure supernatant, and of 4.9 Cq value units (*p* = 0.004) between extracted RNA and non-extracted 1/10 diluted supernatant. The standard RT-qPCR could detect all nine positive mosquito body pools (PM1–PM9), while the direct RT-qPCR could detect four (PM1–PM4) and eight body pools (PM1–PM8), when using non-extracted pure supernatant and non-extracted 1/10 diluted supernatant, respectively (Table 6).

These data highlight the greater efficacy of the 1/10 diluted supernatant compared to the pure supernatant in RVFV direct RT-qPCR experiments. In addition, the mean sensitivity between the pure supernatant and 1/10 diluted supernatant shows a significant difference (*p* = 0.015). For this reason, the use of 1/10 diluted supernatant is recommended in the case of RVFV direct RT-qPCR (Table 7).

### 3.3. Zika Virus

The ZIKV standard and direct RT-qPCR experiments highlighted an average sensitivity difference of 2.4 Cq value units (*p* = 0.004) in Cq values between extracted RNA versus non-extracted pure supernatant, and of 4.5 Cq value units (*p* = 0.002) between extracted RNA versus non-extracted 1/10 diluted supernatant. A mean Cq value difference of 1.9 Cq value units (*p* = 0.007) between non-extracted pure and diluted supernatant was observed, indicating a better sensitivity of the pure supernatant compared to the diluted one, and suggesting its use in the case of the ZIKV direct RT-qPCR experiments (Table 8).

Despite the average difference sensitivity between the standard RT-qPCR and the direct RT-qPCR (pure and 1/10 diluted supernatant), both methodologies could detect all positive mosquito body pools, from the one containing nine infected mosquito bodies (PM1) to the one containing only one infected mosquito body (PM9), showing the high efficiency of the direct RT-qPCR assay in case of ZIKV (Table 9).

## 4. Discussion

Infectious diseases, and particularly, vector-borne infections, are an increasing burden for public health worldwide, constantly expanding into new areas and continents. One essential and strategic way to contain infectious disease outbreaks is early detection before large-scale epidemics develop. However, in low-income countries, infectious disease surveillance is challenging. The monitoring, detection, and prevention of such diseases are negatively impacted by the non-specificity of clinical signs of several arthropod-borne infections, the lack of appropriate surveillance systems, the low availability of diagnostic methods, and the absence of proper public health and laboratory findings [28].

The common gold standard technique used to detect viral pathogens is the polymerase chain reaction (RT-qPCR) although this technique requires the previous isolation and detection of viral DNA/RNA, which is technologically and financially difficult in remote areas and field-laboratory settings [28].

To overcome these limitations, we tried to develop a rapid and simplified assay for the detection of African lineages of CHIKV, RVFV, and ZIKV from experimentally infected mosquito pools by using a direct RT-qPCR assay that would not require prior RNA extraction. The sensitivity and accuracy of direct RT-qPCR from pure supernatant and 1/10 diluted supernatant were evaluated.

In experiments with CHIKV- and RVFV-infected mosquitoes, we observed a wide drop in sensitivity in terms of Cq values between the traditional standard RT-qPCR and the direct RT-qPCR. Particularly, the RVFV standard and direct RT-qPCR experiments resulted in a 8.9 Cq value unit average sensitivity difference between extracted RNA and non-extracted pure supernatant, and in a 4.9 Cq values unit between extracted RNA and non-extracted 1/10 diluted supernatant. The loss of efficacy between the standard and the direct RT-qPCR was also observed for the CHIKV direct RT-qPCR experiments, which highlighted an average difference sensitivity of 5.9 Cq values unit in Cq values between extracted RNA and non-extracted pure supernatant, and of 5.2 Cq values unit between extracted RNA and non-extracted 1/10 diluted supernatant.

Direct RT-qPCR assay using 1/10 diluted supernatant, in the case of CHIKV and RVFV, allowed for the detection of almost all mosquito body pools included in the experiments (from the PM8, containing only two infected mosquito bodies, to the PM1, containing nine infected mosquito bodies), and showed higher accuracy than the pure supernatant, which allowed for the detection of only four mosquito pools over ten. In the case of CHIKV, the standard RT-qPCR could detect seven mosquito body pools (PM1–PM7), while in the direct RT-qPCR experiments, four (PM1–PM4) and six pools (PM1–PM6) were detected, when using non-extracted pure supernatant and 1:10 diluted supernatant, respectively. In both the CHIKV and RVFV experiments, the 1:10 diluted supernatant yielded better results. This better sensitivity with the 1/10 diluted supernatant could be explained by the fact that there are fewer PCR inhibitors in the diluted supernatant.

In ZIKV experiments, direct qRT-PCR allowed for viral detection in all positive mosquito body pools (PM1–PM9). Despite a general loss of sensitivity observed when using direct supernatant compared to extracted RNA, both standard and direct RT-qPCR could detect all positive mosquito body pools (PM1–PM9), confirming the efficacy of the technique. Importantly, following a statistical mean difference observed between non-extracted pure supernatant and 1/10 diluted supernatant, and the higher sensitivity of the pure supernatant compared to the diluted one, the use of pure supernatant is suggested when testing ZIKV.

Compared to the standard RT-qPCR assay, the direct RT-qPCR detected 85.71%, 89%, and 100% of samples with an average difference of about 5.2, 4.9, and 2.4 Cq values unit in the case of CHIKV, RVFV, and ZIKV, respectively. There might be multiple reasons leading to the difference in sensitivity and accuracy of the direct RT-qPCR assays observed for an alphavirus, a phlebovirus, and a flavivirus. A possible explanation could be the varying complexity of the virion structures, with flaviviruses potentially being the less structured and therefore the easiest to detect using this method. (i) The CHIKV has a complex icosahedral structure, constituted by a nucleocapsid core surrounded by a lipid envelope, into which an icosahedral array of glycoproteins is embedded [12,13], (ii) the RVFV is characterized by a lipid bilayer containing two viral glycoproteins enveloping a viral genome helically wrapped in nucleocapsid proteins [20], and (iii) the ZIKV is constituted by a lipid membrane protein bilayer, with the outer surface tiled with a coat of tightly packed envelope proteins in an icosahedral-like symmetry, with the capsid protein forming the innermost layer [14]. Other reasons could explain the slight drop in sensitivity, such as the non-lysis of the particles contained in the supernatant, or the presence of hemoglobin [45] or nucleases (RNase, DNase) [46], which might inhibit the direct RT-PCR. Moreover, the absence of protective agents, such as carrier RNA contained in the QIAamp viral RNA kit buffers, could explain this difference between direct RT-qPCR and standard RT-qPCR.

Despite a general loss of sensitivity and accuracy, the direct RT-qPCR might nevertheless be considered an efficient technique for detection in mosquito pools in field condition experiments when prior RNA extraction is difficult to perform. This technique is an innovative, simple, fast, and alternative workflow for the molecular detection of infectious pathogens, for use in field-laboratory settings during surveillance work for outbreak management. As the best results were obtained with the ZIKV, the direct RT-qPCR with supernatant could be used on the field-caught mosquitoes during a ZIKV outbreak coupled with a mobile laboratory platform as it was already described [47,48]. Briefly, after identification, the field-caught mosquitoes will be grounded in a gloves box and the homogenates will be clarified with centrifugation to obtain the supernatant. Then, the RT-qPCR mix could be prepared and the supernatant will be added to the glovebox. Finally, the direct RT-qPCR could be performed in a portable thermocycler, such as the MIC (Bio Molecular Systems, Upper Coomera, QLD, Australia).

Additionally, it would be wise to use the tandem toehold-mediated displacement reactions (tTMDR) technique on pure or diluted supernatants to improve the performance of our test. The DNA tetrahedron limits the freedom of the single-strand template DNA, thus providing a rigid platform to facilitate the tTMDR amplification. In the first step of the TMDR process, the target RNA is annealed to the complementary DNA sequence via the first toehold, simultaneously displacing the Protector DNA and restoring the labeled DNA fluorescence. In the next TMDR step, Capture DNA displaces the target RNA via the second toehold. Accordingly, the target RNA can be reused for the first TMDR process with another DNA tetrahedron, thus forming an amplification loop that enhances the fluorescence signal with each overall cycle. By the use a of single molecule detection technique, 0.1 attomolar target RNA could be detected. Indeed, the tTMDR was tested on the Dengue virus and was able to detect six copies of RNA per sample [49]. Thus, by using this technique on the supernatants (diluted or not) of the Zika virus, a flavivirus similar to the Dengue virus, with which we obtained the best yields, could be an added value for the rapid detection of this virus.

Furthermore, we expect to test the capacity of RT-LAMP (Reverse Transcription Loop-mediated Isothermal Amplification) to detect our viruses of interest in non-extracted mosquito supernatant.

Direct amplification without prior RNA extraction can allow for early pathogen detection in remote and underdeveloped areas to combat outbreaks and virus spillover more cheaply and simply. Furthermore, this technique might be of fundamental importance during an emergency characterized by global shortage if the supply of viral NA extraction kits is disrupted, as observed during the ongoing COVID-19 pandemic.

In perspective, we suggest performing and completing similar experiments with the WNV, YFV, and Usutu (USUV), and trying to add a simple detergent to the medium to increase the accuracy of the direct PCR assay.

## Figures and Tables

**Table 1:** Viral strains used in this study.

Virus	Strain	Source	Year	Location	Passage History	Lineage	Viral Titer (PFU/mL)
CHIKV	S27	*Homo sapiens*	1953	Tanzania	P8	East Africa	8 × 10^7^
ZIKV	ArD275569	*Aedes leptocephalus*	2017	Senegal	P7	West Africa	1.5 × 10^7^
RVFV	ArD141967	*Culex poicilipes*	2000	Mauritania	P5	West Africa	5 × 10^7^

**Table 2:** Primers and probes used in this study.

Virus	Primers/Probes	Sequences 5′→3′
CHIKV [42]	Forward primer	AAGCTYCGCGTCCTTTACCAAG
	Reverse primer	CCAAATTGTCCYGGTCTTCCT
	Probe	FAM-CCAATGTCYTCMGCCTGGACACCTTT-BBQ
RVFV [43]	Forward primer	TGCCACGAGTYAGAGCCA
	Reverse primer	GTGGGTCCGAGAGTYTGC
	Probe	FAM-TCCTTCTCCCAGTCAGCCCCAC-BBQ
ZIKV [44]	Forward primer	AARTACACATACCARAACAAAGTG GT
	Reverse primer	TCCRCTCCCYCTYTGGTCTTG
	Probe	FAM-CTYAGACCAGCTGAAR-BBQ

FAM, 6-carboxyfluorescein; BBQ, BlackBerry Quencher. +LNA-Nucleotide

**Table 3:** Mosquito body pools.

	CHIKV S27		RVFV ArD141967		ZIKV ARD275569	
	Infected	Non-Infected	Infected	Non-Infected	Infected	Non-Infected
PM1	8	0	9	0	9	0
PM2	7	1	8	1	8	1
PM3	6	2	7	2	7	2
PM4	5	3	6	3	6	3
PM5	4	4	5	4	5	4
PM6	3	5	4	5	4	5
PM7	2	6	3	6	3	6
PM8	1	7	2	7	2	7
PM9	0	8	1	8	1	8
PM10	NA	NA	0	9	0	9

PM = Pools of mosquito bodies and NA = not applicable. The numbers in the table represent the number of mosquito bodies in each pool of mosquito bodies.

**Table 4:** Threshold cycle (Cq) values using standard RT-qPCR (extracted viral RNA) versus direct RT-qPCR (pure or diluted supernatant) for CHIKV detection.

	Extracted Viral RNA (±SD)	Pure The Supernatant (±SD)	Diluted The Supernatant (±SD)
PM1	22.5 (2.30)	29.9 (0.41)	30.6 (0.07)
PM2	27.4 (0.34)	31.5 (0.03)	33.8 (0.06)
PM3	24.3 (0.71)	30.6 (0.71)	31.9 (0.20)
PM4	21.6 (0.21)	32.2 (3.84)	29.6 (0.08)
PM5	27.7 (0.53)	N/A = 40	34.4 (0.69)
PM6	31.1 (1.30)	N/A = 40	36.9 (1.14)
PM7	30.8 (0.38)	N/A = 40	N/A = 40
PM8	N/A = 40	N/A = 40	N/A = 40
PM9	N/A = 40	N/A = 40	N/A = 40

PM = Pools of mosquito bodies. Numbers in the tables represent the RT-PCR cycle threshold values, the number in the bracket represents the ± standard deviation value, N/A means that viral RNA were not detected. Diluted supernatant means 1/10 dilution.

**Table 5:** Direct RT-PCR test parameters for CHIKV.

	Pure Supernatant	Diluted Supernatant
R^2^	0.77	0.92
CI [95%]	[0.29–0.94]	[0.71–0.98]
Sensitivity	100.00%	100.00%
Specificity	20.00%	33.33%
Accuracy	55.56%	77.78%

Note: R^2^ is the coefficient of determination. This coefficient is the square of the Pearson correlation coefficient. The sensitivity, specificity, and accuracy are relative to the standard RT-qPCR results.

**Table 6:** RVFV standard RT-qPCR (RNA) versus direct RT-qPCR (pure supernatant and 1/10 diluted supernatant).

	Extracted Viral RNA (±SD)	Pure Supernatant (±SD)	Diluted Supernatant (±SD)
PM1	31.1 (0.45)	38.9 (0.89)	38.9 (0.45)
PM2	27.9 (0.46)	36.1 (1.20)	30.4 (0.29)
PM3	25.8 (0.10)	32.6 (1.54)	29.9 (0.45)
PM4	27.7 (0.30)	36.5 (3.16)	30.7 (0.05)
PM5	27.3 (0.16)	N/A = 40	30.3 (0.16)
PM6	31.3 (0.34)	N/A = 40	37.3 (0.70)
PM7	24.4 (3.03)	N/A = 40	31.9 (0.38)
PM8	27.8 (0.13)	N/A = 40	34.5 (0.54)
PM9	32.0 (0.04)	N/A = 40	N/A = 40
PM10	N/A = 40	N/A = 40	N/A = 40

PM= Pools of mosquito bodies. Numbers in the tables represent the RT-PCR cycle threshold values, the number in the bracket represents the ± standard deviation value and N/A means that viral RNA were not detected. Diluted supernatant means 1/10 dilution.

**Table 7:** Direct RT-PCR test parameters for RVFV.

	Pure Supernatant	Diluted Supernatant
R^2^	0.38	0.80
CI [95%]	[0.32–0.81]	[0.35–0.95]
Sensitivity	100.00%	100.00%
Specificity	20.00%	50.00%
Accuracy	60.00%	90.00%

Note: R^2^ is the coefficient of determination, this coefficient is the square of the Pearson correlation coefficient. The sensitivity, specificity, and accuracy are relative to the standard RT-qPCR results.

**Table 8:** ZIKV standard RT-qPCR (RNA) versus direct RT-qPCR (pure supernatant and 1/10 diluted supernatant).

	RNA (±SD)	Pure Supernatant (±SD)	Diluted Supernatant (±SD)
PM1	24.1 (0.59)	25.9 (0.33)	28.7 (0.32)
PM2	20.7 (0.08)	23.9 (0.15)	26.4 (0.17)
PM3	22.5 (0.26)	24.8 (0.15)	27.6 (0.02)
PM4	20.8 (0.52)	25.0 (0.21)	27.6 (0.05)
PM5	24.5 (0.17)	26.7 (0.31)	29.2 (0.32)
PM6	22.4 (0.13)	24.9 (0.48)	28.0 (0.24)
PM7	23.6 (0.27)	25.9 (0.29)	28.8 (0.12)
PM8	23.8 (0.18)	26.7 (0.11)	29.5 (0.15)
PM9	25.2 (0.35)	27.7 (0.13)	29.5 (0.53)
PM10	N/A = 40	N/A = 40	N/A =40

PM = Pools of mosquito bodies. Numbers in the tables represent the RT-PCR cycle threshold values, the number in the bracket represents the ± standard deviation value and N/A means that viral RNA were not detected. Diluted supernatant means 1/10 dilution.

**Table 9:** Direct RT-PCR test parameters for ZIKV.

	Pure Supernatant	Diluted Supernatant
R^2^	0.99	0.99
CI [95%]	[0.97–0.99]	[0.97–0.99]
Sensitivity	100.00%	100.00%
Specificity	100.00%	100.00%
Accuracy	100.00%	100.00%

Note: R^2^ is the coefficient of determination, this coefficient is the square of the Pearson correlation coefficient. the sensitivity, specificity, and accuracy are relative to the standard RT-qPCR results.

## Data Availability

Data are contained within the article.
